# Management of Aggressive Recurrent Meningioma Using a Combined Transfacial-Pterional Approach

**DOI:** 10.7759/cureus.91531

**Published:** 2025-09-03

**Authors:** Alan Ferrufino-Mejia, Héctor A Rodríguez-Rubio, Shirley Rocío Chavarría-Mejía, Dara Lizeth Torres-Rodríguez, Roy Ferrufino-Mejia

**Affiliations:** 1 Neurological Surgery, Angeles Metropolitan Hospital, Mexico City, MEX; 2 Neurological Surgery, National Medical Center 20 de Noviembre Institute of Security and Social Services for State Workers, Mexico City, MEX; 3 Neuroanesthesiology, Mexican Institute of Social Security XXI Century National Medical Center, Mexico City, MEX; 4 Neuroendovascular Therapy, Juárez Hospital of Mexico, Mexico City, MEX

**Keywords:** combined craniofacial approach, extracranial extension, meningothelial meningioma, skull base surgery, sphenoid wing meningioma

## Abstract

Skull base meningiomas, although histologically benign, can exhibit locally aggressive behavior, particularly when arising from the sphenoid wing. Their potential to infiltrate bone and extend extracranially into the orbit, infratemporal fossa, and facial soft tissues significantly increases surgical complexity and recurrence risk. Complete resection in these cases requires wide anatomical exposure and multidisciplinary planning to preserve critical neurovascular structures while achieving oncologic control. We present the case of a woman with a recurrent sphenoid wing meningioma extending into the middle cranial fossa, orbit, masticator space, and premaxillary region. A single-stage combined transfacial (Weber-Ferguson) and extended pterional approach was performed, allowing for gross total resection with preservation of function and anatomy. Histopathology confirmed a WHO Grade I meningothelial meningioma. Postoperative imaging demonstrated complete resection, and the patient remains neurologically intact under continued surveillance. This case highlights the role of tailored craniofacial approaches in managing complex, multicompartmental skull base meningiomas with extracranial extension.

## Introduction

Meningiomas are the second most common primary intracranial neoplasms, accounting for 15-20% of central nervous system tumors [1]. Although generally benign and slow-growing, meningiomas involving the skull base pose distinct surgical challenges [2]. Their tendency to invade dura, bone, and foraminal structures complicates complete resection and increases the risk of recurrence. Even after gross total resection, recurrence rates reach 7-10% at five years and up to 22% at 10 years [3,4]; subtotal resections carry significantly higher rates, exceeding 70% in a decade [5]. Sphenoid wing meningiomas, especially those arising medially, can extend extracranially into the orbit, infratemporal fossa, pterygopalatine space, or paranasal sinuses [6]. These recurrences demand complex craniofacial approaches, often requiring collaboration between neurosurgeons and head and neck surgeons. Although newer skull base techniques improve access and outcomes, gross total resection remains the cornerstone of treatment [7]. We present a rare case of a recurrent meningothelial meningioma with extensive craniofacial extension, managed through a single-stage combined pterional and transfacial (Weber-Ferguson) approach [8]. This case highlights the surgical rationale and technical nuances necessary to address aggressive skull base meningiomas with extracranial involvement.

## Case presentation

A 49-year-old woman with a history of type 2 diabetes mellitus and previous oncologic interventions presented with progressive craniofacial and neurological symptoms. Years earlier, she had undergone an excision of a left temporoorbital mass initially diagnosed as squamous cell carcinoma, and had been treated with adjuvant radiotherapy. Subsequent imaging revealed abnormal uptake in the orbital and sphenoidal regions, and surgical resection confirmed the presence of an atypical meningioma.

In the two to three years preceding her current admission, she experienced new otologic, nasal, and neurological manifestations. The key presenting symptoms, physical examination, and neurological findings are summarized in Table 1.

**Table 1:** Summary of key clinical features at presentation OS: Oculus Sinister or left eye. The table outlines the patient’s main presenting symptoms, physical examination findings, and neurological deficits. Quantitative details, including seizure frequency, proptosis measurement, and Snellen visual acuity, are provided to facilitate comparison with similar cases.

Domain	Findings
Presenting symptoms	Left-sided sensorineural hearing loss (sudden onset, ~2-3 years prior); generalized tonic-clonic seizures (frequency: 2-3 per month, controlled with antiepileptics); unilateral nasal obstruction; paresthesia of the upper lip; progressive proptosis; gradual decline in left visual acuity.
Physical examination	Left facial asymmetry; axial proptosis of 4 mm (Hertel exophthalmometer, compared with right eye); hemorrhagic edema of the posterior maxillary vestibule.
Neurological findings	Hypoesthesia in the maxillary nerve (V2) distribution; decreased visual acuity OS to 20/80 Snellen, consistent with optic nerve (II) involvement; subtle orbicularis oris asymmetry and paresis of the frontal branch of the facial nerve (House–Brackmann grade I); limitation of ocular motility in supraversion and horizontal gaze.

**Figure 1:**
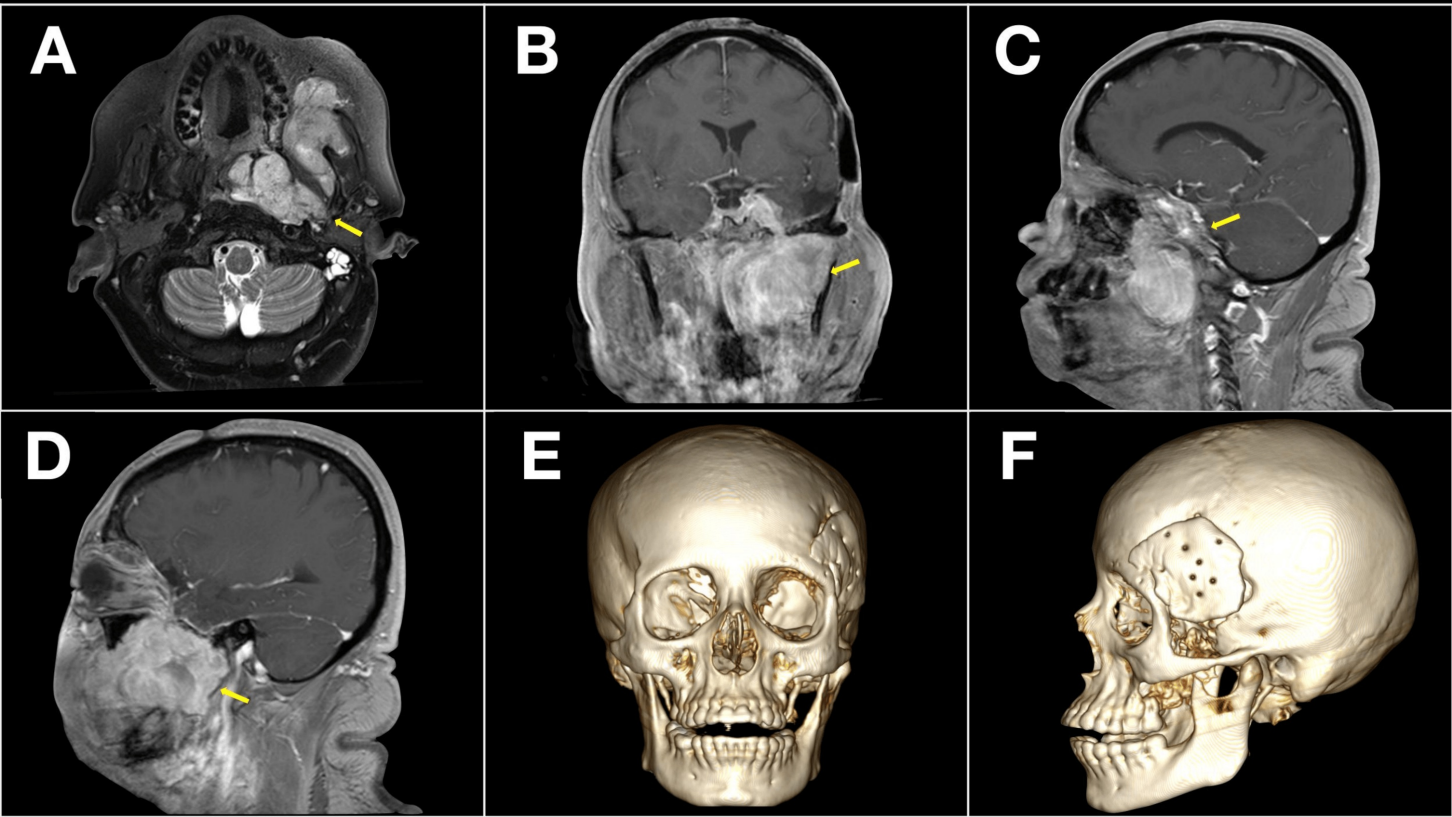
Preoperative MRI and 3D reconstruction Yellow arrows indicate the relevant findings in all panels. (A) Axial T2-weighted MRI showing a multilobulated mass extending from the left middle cranial fossa into the orbit, masticator space, and premaxillary region; (B) Coronal post-contrast T1-weighted MRI demonstrating heterogeneous enhancement with bone remodeling and soft tissue infiltration; (C) Sagittal MRI revealing anterior orbital and maxillofacial extension; (D) Lateral sagittal view highlighting skull base involvement and orbital displacement; (E) Frontal 3D CT reconstruction showing distortion of the zygomatic arch, lateral orbital rim, and anterior maxilla; (F) Lateral 3D reconstruction illustrating midfacial and anterior cranial base alterations.

Magnetic resonance imaging (MRI) demonstrated a multilobulated, contrast-enhancing mass occupying the middle cranial fossa, orbit, premaxillary soft tissues, and masticator space. The tumor exhibited aggressive extracranial extension, bone remodeling, and displacement of orbital contents, raising suspicion for atypical meningioma or hemangiopericytoma (Figures 1A-1D).

A preoperative 3D reconstruction confirmed osseous distortion in the zygomatic arch and orbital rim (Figures 1E-1F).

**Figure 2:**
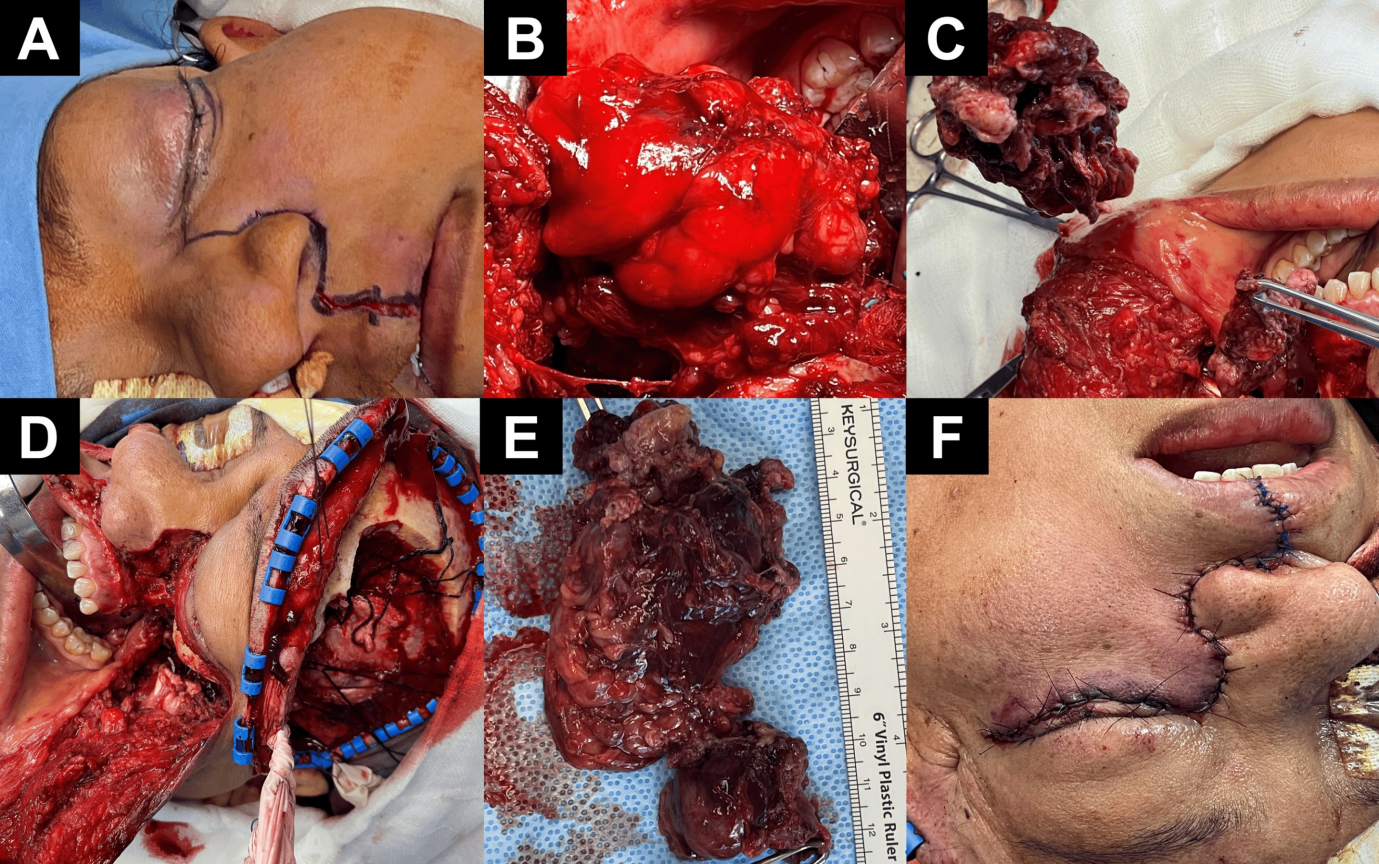
Intraoperative sequence of combined transfacial and transcranial approach (A) Preoperative marking of the Weber-Ferguson incision delineating transfacial access to the midface and orbit; (B) Initial exposure revealing a multilobulated, highly vascularized mass infiltrating the maxillary and masticator spaces; (C) Mobilization of extracranial tumor components through en bloc dissection of involved soft tissues; (D) Combined exposure following osteotomies of the zygomatic arch and lateral orbital rim, providing access to the skull base; (E) Resected specimen showing multiple lobules with firm consistency and hemorrhagic surface, measuring approximately 9 cm; (F) Immediate postoperative closure with layered suturing and anatomical alignment of facial incision.

A single-stage combined craniofacial approach was performed. A left-sided transfacial Weber-Ferguson incision (Figure 2A) allowed wide exposure of the midface and orbit. Subperiosteal dissection revealed a highly vascularized, multilobulated tumor involving the maxillary sinus, pterygopalatine and masticator spaces, and lateral orbital wall (Figures 2B-2C).

En bloc mobilization of the tumor lobules was achieved through the sequential dissection of infiltrated soft tissues and bone. Simultaneously, a left pterional craniotomy was extended toward the lateral orbital rim and zygomatic root. Osteotomies enabled temporary mobilization of the zygomatic arch and orbital roof, granting access to the skull base and infratemporal fossa (Figure 2D). Meticulous microdissection permitted complete tumor excision while preserving key neurovascular structures. The resected specimen consisted of a multilobulated, firm mass measuring approximately 9 cm in greatest dimension, consistent with meningioma (Figure 2E). Facial reconstruction and layered closure achieved aesthetic alignment with minimal tension (Figure 2F).

**Figure 3:**
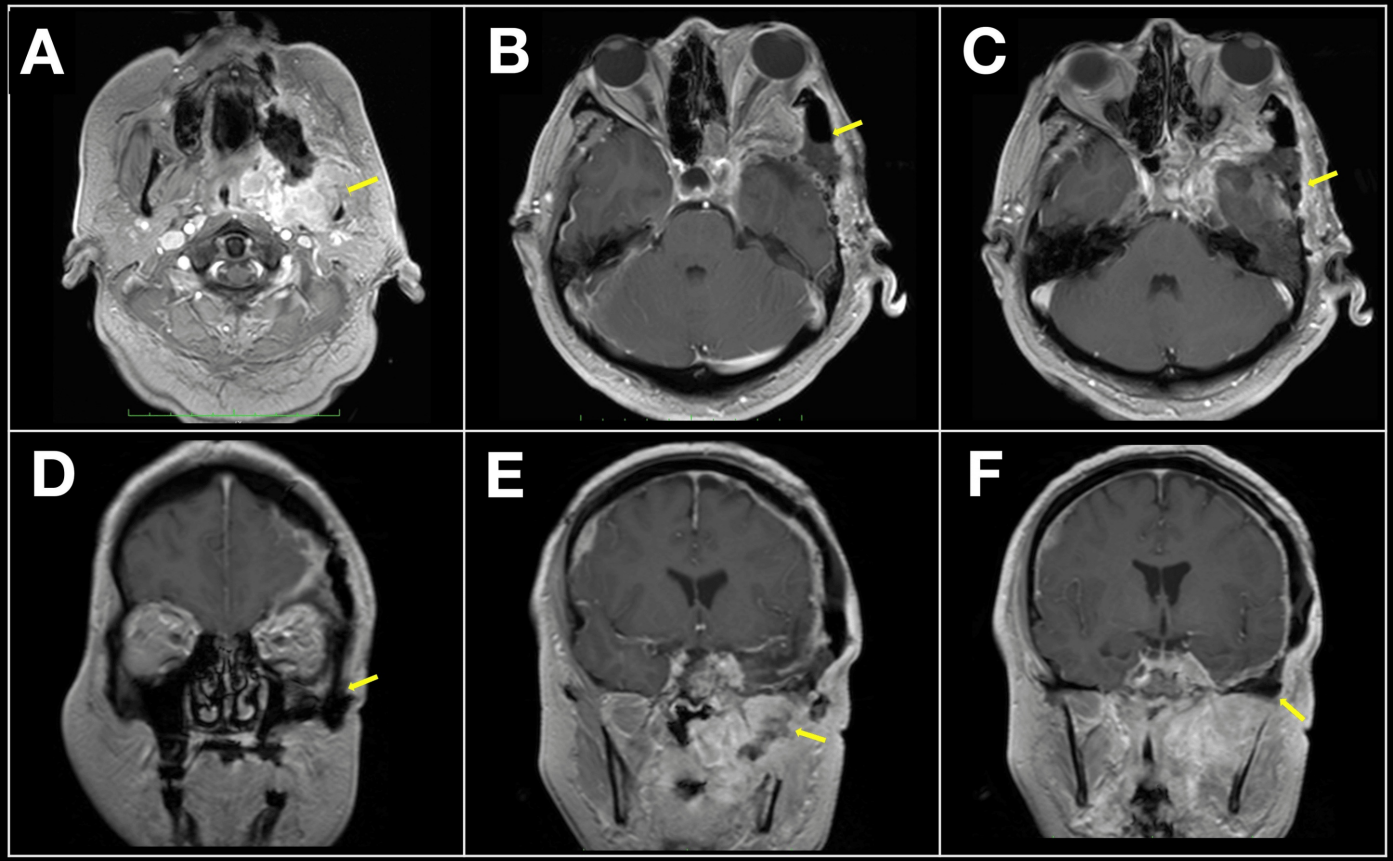
Postoperative MRI demonstrating gross total resection of recurrent sphenoid wing meningioma Yellow arrows indicate the relevant findings in all panels. (A) Axial T2-weighted MRI confirming resolution of extracranial extension with preserved surrounding structures; (B, C) Axial T1-weighted post-contrast views showing no residual enhancing tissue in the orbit, masticator space, or middle cranial fossa; (D) Coronal T1-weighted image illustrating decompression of the orbit and normalization of midfacial contours; (E,F) Coronal post-contrast views confirming complete resection with anatomical restoration of the skull base and absence of residual tumor.

Postoperative imaging confirmed gross total resection with no residual contrast-enhancing tissue and adequate anatomical restoration (Figures 3A-3F).

Histopathological analysis revealed a WHO Grade I meningothelial meningioma [9]. Clinical follow-up consisted of regular neurosurgical evaluations and serial MRI studies, with the patient remaining neurologically stable and without radiological evidence of recurrence at 12 months postoperatively.

## Discussion

Meningiomas involving the sphenoid wing represent a distinct neurosurgical challenge, particularly when demonstrating extracranial extension into facial compartments. Although typically classified as WHO Grade I tumors, these lesions can exhibit biologically aggressive behavior, especially in the setting of recurrence [10]. Maroon et al. reported recurrence rates of 35% to 50% in sphenoid wing meningiomas with orbital extension, highlighting the need for early, radical intervention with careful attention to the orbit, cavernous sinus, and adjacent neurovascular structures [11]. In the present case, the meningioma demonstrated extensive invasion into the middle cranial fossa, orbit, premaxillary soft tissues, and masticator space. This complex anatomic distribution made limited-access approaches inadequate. Therefore, a combined extended pterional and transfacial Weber-Ferguson approach was employed in a single-stage procedure, providing multidirectional access to both intra- and extracranial compartments. The extended pterional craniotomy allowed subtemporal and lateral orbital dissection, while the Weber-Ferguson incision offered wide exposure to the midface, zygomatic arch, orbit, and infratemporal fossa. This dual exposure enabled precise microsurgical dissection, preservation of neurovascular structures, and en bloc mobilization of the tumor lobules across involved planes [12].

Kim et al. previously demonstrated that transfacial access offers superior exposure to anterior and lateral skull base regions when conventional transcranial or endoscopic methods are insufficient [13]. Similarly, Iaconetta et al. highlighted that the progression of extracranial meningioma frequently occurs through foramina such as the foramen rotundum or the pterygopalatine canal, necessitating aggressive resection of both soft tissue and hyperostotic bone [14]. In our case, osteotomies of the zygomatic arch and lateral orbital rim, performed as part of the combined approach, were essential in achieving surgical clearance and facilitating direct access to deep facial and skull base structures.

Among transfacial techniques, the Weber-Ferguson incision remains a cornerstone for extensive craniofacial tumors involving the orbitomaxillary and infratemporal regions. While associated with a central facial scar, it provides unmatched direct exposure, allowing for meticulous tumor resection and controlled hemostasis [15]. Alternative approaches such as transoral or transnasal corridors may offer cosmetic advantages, but often lack the depth and lateral access required for large, vascular, or recurrent lesions [16]. Nonaka et al. reinforced the need for individualized, anatomy-driven planning in skull base surgery, particularly in the context of vascular involvement or recurrence [17]. While their case required a transcavernous-infratemporal approach with bypass, our patient benefited from a single-stage combined approach that minimized anesthetic exposure and enabled coordinated tumor resection and reconstruction. As emphasized by Rao et al., recurrence risk remains elevated in residual post-radiotherapy lesions. Achieving gross total resection remains critical [18]. Our case supports the paradigm that aggressive, anatomically informed strategies, particularly those combining extended transcranial and transfacial exposure, optimize oncologic outcomes in complex skull base meningiomas with a craniofacial extension.

## Conclusions

Meningiomas with extracranial extension to facial structures represent a clinically challenging entity that requires extensive and personalized surgical approaches. The combination of transfacial and transcranial access allows adequate exposure of anatomically complex regions, favoring complete tumor resection and reducing the risk of recurrence. Performing the procedure in a single surgical stage optimizes patient recovery and minimizes morbidity associated with sequential interventions. Importantly, this case illustrates that gross total resection with preservation of neurological and functional outcomes is achievable even in recurrent sphenoid wing meningiomas with extracranial extension. Given their recurrent potential, close monitoring through regular imaging remains essential, while multidisciplinary planning and radical resection continue to be key pillars in the successful management of meningiomas with craniofacial extensions.
